# Early detection of Ebola virus proteins in peripheral blood mononuclear cells from infected mice

**DOI:** 10.1186/s12014-020-09273-y

**Published:** 2020-03-17

**Authors:** Michael D. Ward, Tara Kenny, Ernie Bruggeman, Christopher D. Kane, Courtney L. Morrell, Molly M. Kane, Sandra Bixler, Sarah L. Grady, Rachel S. Quizon, Mekbib Astatke, Lisa H. Cazares

**Affiliations:** 1 Systems and Stuctural Biology Division, Protein Sciences Branch, U.S. Army Medical Research Institute of Infectious Diseases, Frederick, MD USA; 2 Therapeutic Development Center, U.S. Army Medical Research Institute of Infectious Diseases, Frederick, MD USA; 3 Applied Biological Sciences, The Johns Hopkins University Applied Physics Laboratory, 11100 Johns Hopkins Road, Laurel, MD USA

**Keywords:** Ebola virus, Quantitative proteomics, Mouse adapted Ebola virus, Diagnostics

## Abstract

**Background:**

Detection of viral ribo-nucleic acid (RNA) via real-time polymerase chain reaction (RT-PCR) is the gold standard for the detection of Ebola virus (EBOV) during acute infection. However, the earliest window for viral RNA detection in blood samples is 48–72 h post-onset of symptoms. Therefore, efforts to develop additional orthogonal assays using complementary immunological and serological technologies are still needed to provide simplified methodology for field diagnostics. Furthermore, unlike RT-PCR tests, immunoassays that target viral proteins and/or early host responses are less susceptible to sequence erosion due to viral genetic drift. Although virus is shed into the bloodstream from infected cells, the wide dynamic range of proteins in blood plasma makes this a difficult sample matrix for the detection of low-abundant viral proteins. We hypothesized that the isolation of peripheral blood mononuclear cells (PBMCs), which are the first cellular targets of the Ebola virus (EBOV), may provide an enriched source of viral proteins.

**Methods:**

A mouse infection model that employs a mouse-adapted EBOV (MaEBOV) was chosen as a proof-of-principal experimental paradigm to determine if viral proteins present in PBMCs can help diagnose EBOV infection pre-symptomatically. We employed a liquid chromatography coupled with tandem mass spectrometry (LC–MS/MS) platform to provide both high sensitivity and specificity for the detection and relative quantitation of viral proteins in PBMCs collected during MaEBOV infection. Blood samples pooled from animals at the post-infection time-points were used to determine the viral load by RT-PCR and purify PBMCs.

**Results:**

Using quantitative LC-MS/MS, we detected two EBOV proteins (vp40 and nucleoprotein) in samples collected on Day 2 post-infection, which was also the first day of detectable viremia via RT-PCR. These results were confirmed via western blot which was performed on identical PBMC lysates from each post-infection time point.

**Conclusions:**

While mass spectrometry is not currently amenable to field diagnostics, these results suggest that viral protein enrichment in PBMCs in tandem with highly sensitive immunoassays platforms, could lead to the development of a rapid, high-throughput diagnostic platform for pre-symptomatic detection of EBOV infection.

## Background

Ebola virus (EBOV) and Marburg virus (MARV) are members of the *Filoviridae* family of highly virulent human pathogens that cause severe illness with high fatality rates and for which there are no available FDA-approved vaccines or therapeutics. Once infected, the incubation period of Ebola virus disease (EVD) in humans can vary from 2 to 21 days, and it presents initially with common symptoms such as fever, fatigue, muscle pain, headache, and sore throat. Real-time polymerase chain reaction (RT-PCR) is the gold standard for the detection of EBOV during acute infection in outbreak settings, but the earliest window for viral genomic RNA detection in blood samples is roughly 48–72 h post-onset of symptoms [1, 2]. For RT-PCR based diagnosis of EVD, RNA is first extracted from whole blood, and extraction requires several steps, including sample collection and inactivation, viral RNA extraction, reverse transcription and cDNA amplification. RNA-based diagnostic approaches can be difficult to deploy for point of care settings, especially in resource limited environments and field settings where electrical powered lab equipment may not be available. In addition, the need to store RNA samples at − 80 °C to prevent degradation further decreases the utility of RT-PCR assays for EVD diagnostic applications in austere field settings. Contamination of viral RNA samples during processing for RT-PCR is also common, therefore careful attention to decontamination protocols at Ebola treatment centers is required to minimize false-positive results [3]. Current commercially available RT-PCR and immunoassay tests for EVD diagnosis are limited for clinical conditions after onset of clinical symptoms [4] where the likelihood of the virus being transmitted is significantly increased and the outcome of current medical interventions are less effective.

The genome of EBOV is linear, (−) ssRNA and encodes for seven genes which produce 9 proteins: a nucleoprotein (NP), a polymerase cofactor (VP35), a matrix protein (VP40), a transmembrane glycoprotein (GP), 2 truncated secreted versions of GP (sGP, and ssGP), a transcriptional activator (VP30), a viral envelope-associated protein (VP24) and a RNA-dependent RNA polymerase (L) [5]. A number of RT-PCR assays have been developed to target EBOV genes, including the two most abundant constituent parts of the Ebola virion, VP40 and GP [2]. Unfortunately, the low fidelity of the EBOV L protein leads to high genetic drift, meaning that for every new EBOV outbreak, there is the potential for reduced RT-PCR assay sensitivity and specificity due sequence variations between viral strains [6, 7]. Although current WHO and CDC diagnostic protocols recommend RT-PCR as the predominant diagnostic method for EVD [8], efforts to develop additional orthogonal assays using complementary immunological and serological technologies to provide confirmatory diagnostic assays are still needed. Unlike RT-PCR tests, immunoassays that target viral antigens and/or early immunoglobulin M (IgM) host responses are less susceptible to sequence erosion due to genetic drift. The efficacy of an immunoassay that has been approved by WHO for EVD diagnosis; ReEBOV Antigen Rapid Test (Corgenix, Broomfield, CO, USA), was evaluated using whole blood or plasma samples during the 2014 EBOV outbreak [9]. The assay time to result for ReEBOV kit was approximately 20 min, compared to 90 min to 6 h for RT-PCR assays; unfortunately, ReEBOV was shown to be insensitive for diagnosing EVD at acute stage of infection. However, the simplicity (no sample extraction) and rapid sample-to-result features of immunoassay platforms make them attractive candidates for future EBOV diagnostic assays to be developed for field use. Towards this end, identifying viral protein antigens in whole blood or other clinical bio-specimens can provide novel targets for these types of assays.

Non-human primate (NHP) models of EBOV infection have provided the most informative data related to the pathology of EVD and recapitulate many features of the disease in humans. These models have demonstrated that macrophages and dendritic-like cells are the primary targets for EBOV infection [10–12]. Although virus is shed into the bloodstream from these infected cells, the wide dynamic range of proteins in serum and plasma makes them difficult sample matrices for the detection of low-abundance viral proteins. Therefore, the isolation of peripheral blood mononuclear cells (PBMCs) that comprises lymphocytes (T cells, B cells, and NK cells), monocytes, and dendritic cells, may provide an enriched source of viral proteins, especially at the earliest stages of EBOV infection. The cost associated with NHP studies and the difficulty to standardize sampling times con-currently with ongoing vaccine and therapeutic studies makes performing initial studies to collect NHP PBMC samples for viral antigen detection somewhat prohibitive. Therefore, given the similarities of the natural histories and disease manifestation of EBOV infection in mice and non-human primates [13, 14], the mouse-model of a mouse-adapted Ebola (MaEBOV) infection was chosen as a proof-of-principal experimental paradigm to determine if viral proteins present in PBMC populations can help diagnose EBOV infection pre-symptomatically. This model allowed us to explore the potential of viral protein detection at the earliest stages of EVD by interrogating PBMC lysates. For this study we employed a semi-quantitative liquid chromatography coupled with tandem mass spectrometry (LC–MS/MS) platform to provide both high sensitivity and specificity for the detection and relative quantitation of viral proteins in PBMC collected during MaEBOV infection.

## Materials and methods

### Animal use and ethics statement

The facility where this research was conducted is accredited by the Association for Assessment and Accreditation of Laboratory Animal Care, International and adheres to principles stated in the Guide for the Care and Use of Laboratory Animals, National Research Council, 2011. Research was conducted under Institutional Animal Care and Use Committee (IACUC) approved protocols in compliance with the Animal Welfare Act, PHS Policy, and other Federal statutes and regulations relating to animals and experiments involving animals.

### MaEBOV infection

Each experimental cohort consisted of 35 six to twelve-week-old c57Bl/6 mice (Charles River Laboratories) divided into one control (n = 5) and five challenge groups (n = 6) (Table 1). Two independent experimental trials were conducted approximately 2 months apart, using a total of 70 female mice. In order to ensure sufficient cell numbers to perform the analysis, six animals were required for each PI time-point (Days 2, 3, 4 and 5). Laboratory mice were inoculated IP with a target dose of 1000 plaque-forming units (PFU) of MaEBOV in an inoculation volume of 200 µL. All live-virus studies were conducted under maximum BSL-4 containment. Clinical observations were recorded throughout the study starting on the day of virus inoculation. Moribund mice were euthanized according to institution-approved clinical scoring. Blood samples were obtained from anesthetized mice via exsanguination by cardiac puncture in K3-EDTA Greiner Vacuette tubes.

**Table 1:** Experimental design for serial sacrifice

Group	Total dose (PFU)	Sacrifice time-point	Challenge
1 (n = 5)	0	Day 0	PBS
2 (n = 6)	1000	Day 1	IP MaEBOV
3 (n = 6)	1000	Day 2	IP MaEBOV
4 (n = 6)	1000	Day 3	IP MaEBOV
5 (n = 6)	1000	Day 4	IP MaEBOV
6 (n = 6)	1000	Day 5	IP MaEBOV

### Ebov RT-PCR

For quantitative assessment of viral RNA, whole blood was collected using a K3-EDTA Greiner Vacuette tube (or equivalent) and centrifuged at 2500×*g* (± 200) for 10 ± 2 min. To inactivate virus, plasma was treated with three parts (300 μl) TriReagent LS, incubated at least 5min and then samples were transferred to frozen storage (− 60 °C to − 90 °C), until removal for RNA extraction. Carrier RNA and QuantiFast High Concentration Internal Control (Qiagen; Cat #211492) were spiked into the sample before extraction. Viral RNA was eluted in AVE buffer (RNase-free water with 0.04% NaN3: Qiagen). Each extracted RNA sample was tested with the QuantiFast Internal Control RT–PCR RNA Assay (Qiagen; Cat #211452) to evaluate the yield of the spiked QuantiFast High Concentration Internal Control. If the internal control amplified within manufacturer-designated ranges, the test was considered valid and further quantitative analysis of the viral target was performed. RT–PCR was conducted in triplicate on ABI 7500 Fast Dx using primers specific to EBOV GP sequence. For quantitative assessments, averages of the triplicate genomic equivalents (GE) per reaction were determined and multiplied by the appropriate dilution factor to obtain GE mL^−1^ plasma. Standard curves were generated using synthetic RNA. The limit of detection for this assay is 1 × 10^2^ GE/mLof plasma.

### PBMC purification and EBOV inactivation

Blood collected from each group of mice was pooled into a 15 mL conical centrifuge tube and diluted 1:2 in 1× phosphate buffered saline (PBS) for a final volume of approximately 12 mL. The diluted blood was then layered onto 4 mL of Ficoll in Accuspin tubes (12 mL volume), dividing equally between two Accupsin tubes. The tubes were centrifuged at approximately 400×*g* with (no brake). Plasma was removed (~ 300 µL) and transferred to a 2 mL cryovial for storage at − 80 °C. PBMCs in the buffy coat were removed and transferred to a new 15 mL conical tube. Sample volume was increased to 15 mL using 1× PBS and the tubes were centrifuged at approximately 600×*g* with the brake on to pellet the PBMCs. The supernatants were removed and discarded and the cellular material was transferred to a 2 mL cryovial. For cell lysis and EBOV inactivation, the PBMC samples were lysed in 300 µL of RIPA Lysis and Extraction Buffer (Thermo Fisher Scientific; Cat# 89900) treated with HALT Protease Inhibitor Cocktail (Thermo-Fisher Cat# 78430). The samples were vortexed well and kept on ice for 10 min. Samples were then stored at – 80 °C until removed from containment. Total protein concentration of each PBMC lysate was determined using the micro BCA Protein Assay Kit (Thermo Fisher Scientific; Cat# 23225).

### PBMC sample preparation for mass spectrometry

PBMC samples were transferred to a BSL-2 freezer and stored at − 80 °C until processed by iFASP [15]. Briefly, 30 µg of each inactivated PBMC lysate was added to 200 µL 8 M Urea/0.1 M Tris-HCl pH 8.5 and filtered through a Microcon-30 kDa Centrifugal Filter Unit with an Ultracel-30 membrane (Millipore; Cat# MRCF0R030) at 14,000×*g* for 15 min. Filters were washed 3× with 100 mM Tris pH 8.0, and proteins alkylated with 55 mM iodoacetamide followed by digestion with 4 µg Trypsin/Lys-C (Promega, Cat# V5071) overnight at 37 °C. TMT six-Plex labeling (Thermo Fisher Scientific; Cat# 90061) was performed directly on the FASP filters per the manufacturer’s instructions. TMT- labeled digests were then purified by C18 spin column, dried to completion by speed-vac and stored at − 20 °C until analyzed by LC–MS/MS.

### LC–MS/MS TMT analysis and calculation of protein abundances

Sample digests were suspended in 240 µL of 0.1% formic acid. A Dionex 3000 RSLCnano system (Thermo Fisher Scientific) injected 5 µL of each digest onto a pre-column (C18 PepMap 100, 5 µm particle size, 5 mm length × 0.3 mm internal diameter) housed in a 10-port nano switching valve using a flow rate of 10 µL/min. The loading solvent was 0.1% formic acid in HPLC grade water. Peptides were then loaded onto an Easy-Spray analytical column (15 cm × 75 um) packed with PepMap C18, 3 µm particle size, 100A porosity particles (Thermo Fisher Scientific). A 2–38% B gradient elution in 160 min was formed using Pump-A (0.1% formic acid) and pump-B (85% acetonitrile in 0.1% formic acid) at a flow rate of 300 nL/min. The column eluent was connected to an Easy-Spray source (Thermo Fisher Scientific) with an electrospray ionization voltage of 2.2 kV. An Orbitrap Elite MS (Thermo Fisher Scientific) with an ion transfer tube temperature of 300 °C and an S-lens setting of 55% was used to focus the peptides. A top 15 data dependent MS/MS analysis (DDA) method was used to select the top 15 most abundant ions in a 400–1600 amu survey scan (120,000 resolution FWHM at m/z 400) with a full AGC target value of 1 x 10^6^ ions and a maximum injection time of 200 ms. Higher Energy Collisional Dissociation (HCD) MS/MS spectra were acquired at a resolution of 30,000 (FWHM at m/z 400) with an AGC target value of 5 × 10^4^ ions and a maximum injection time of 200 ms. The isolation width for MS/MS HCD fragmentation was set to 2 Daltons. The normalized HCD Collision energy was 40% with an activation time of 0.1 ms and dynamic exclusion duration of 30 s. A parent ion inclusion list containing 35 m/z values corresponding to EBOV peptides was used in targeted runs (Table 2). Additionally, an exclusion list of commonly observed hemoglobin peptides was used to improve sensitivity samples containing lysed red blood cells (see Additional file 1: Table S1). Protein abundances across time points were determined by combining the Tandem Mass Tag-labeled samples at equivalent concentrations and then measuring the relative intensity of each time points’ contribution to the total protein abundance within the fragmentation spectrum. The pre-infection abundance determined from the Day 0 sample was used as a baseline and assigned a value of 1. The ratios of the intensity of the reporter ions associated with the post-infection time points versus the intensity of the reporter ion in the pre-infection sample were acquired as relative peptide abundance (fold change from baseline).

**Table 2:** List of 36 inclusion masses for the four EBOV proteins: VP30, VP40, GP and NP

Protein	Peptide sequence	Target m/z	
		2+	3+
VP30	VEPLTVPPAPK	803.5026	536.0042
	DGHDHHVR	601.3048	401.2056
	LANPTADDFQQEEGPK	1109.5732	740.0513
	EGLGQDQAEPVLEVYQR	1080.5604	720.7093
	DHQLESLTDR	721.8755	481.5861
VP40	VILPTAPPEYMEAIYPVR	1144.6317	763.4236
	IQAIMTSLQDFK	927.0340	618.3584
	QIPIWLPLGVADQK^a^	1018.6191	679.4151
	LGPGIPDHPLRLLR	892.0488	595.0350
	LRPILLPNK	761.5159	508.0130
GP	SEELSFTAVSNR	784.9095	523.6088
	IDQIIHDFVDK^a^	901.0166	601.0135
	SVGLNLEGNGVATDVPSATK	1194.1628	796.4443
NP	ELPQDEQQDQDHTQEAR^a^	1148.5356	766.0262
	ELDHLGLDDQEK^a^	935.5016	624.0035
	LTEAITAASLPK^a^	837.0161	558.3465
	EAATEAEK^a^	653.8664	436.2467
	NEPSGSTSPR	630.8227	420.8842

^a^Observed in EBOV infected mouse PBMCs. 1 PSM of GP detected

### Database search

Acquired MS/MS protein searches were performed with ProteomeDiscoverer 2.2 (Thermo Fisher Scientific) using a *Mus* (TaxID 10090) and *EBOV* (TaxID 128591) subset of the SwissProt_2017_10_25 database containing 25, 103 and 9 sequences respectively. Variable modifications used were TMT 6-plex (N-terminal, K), Carbamyl (KMR), Methyl (DE), Acetyl (K), Deamidated (NQ), and Oxidation (M). Cysteine carbamidomethylation was specified as a constant modification. The false discovery rate (FDR) was set at 0.1% using Posterior Error Probability validation. Only proteins having at least two peptide spectral matches (PSM) were considered. Normalization by total peptide amount was performed using control channel average scaling mode turned on. Mass tolerances were 10 ppm for the MS1 scan and 0.6 Da for all MS/MS scans. Quantitation results were filtered such that only high-confidence/unambiguous PSM’s having MS2 isolation interference values equal to or less than 35% were used.

### Western blot analysis

Western blot assays were performed using a mouse monoclonal antibody (mAb) for VP40 (IBT Bioservices; Mouse anti-EBOV VP40 mAb (3G5) Cat#: 0201-016) and a rabbit polyclonal antibody directed at EBOV NP (IBT Bioservices; Cat# 0301-012). Briefly, PBMC lysates (30 µg total protein) were run under reducing conditions on a 4–12% NuPAGE bis-tris polyacrylamide gel (Thermo Fisher Scientific; Cat# NP0321) and transferred to PVDF membranes. Each blot was blocked overnight with blocking buffer in PBS (Thermo Fisher Scientific; Cat# 37572) and then incubated with primary antibody against VP40 (1:500) or NP (1:1000) overnight at 4 °C on a rocking platform. After washing 3× with PBS + 0.1% Tween-20 (PBST) for 5 min, secondary antibody (goat anti-mouse or goat anti-rabbit IRDye 680 labelled (LICOR Biosciences; Cat #s 926-68070 and 926-68071) was added and the blots were incubated for an additional hour. The blots were again washed 3X with PBST, and then stored in PBS until visualization with an Odyssey infrared imaging system (LI-COR Biosciences; model # 9210).

## Results

### Sample collection and processing

**Fig. 1:**
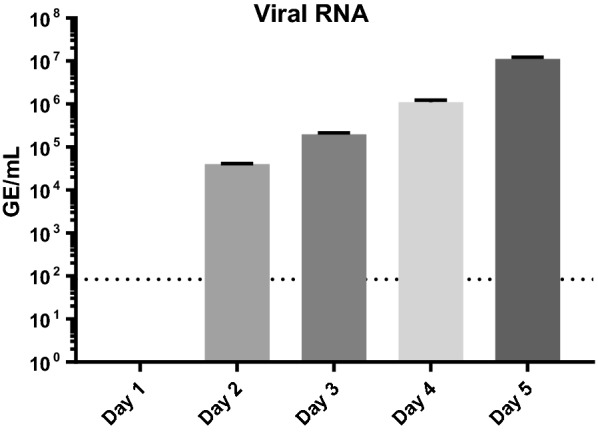
Viral titers during EBOV infection in mice from two independent cohorts. RNA was extracted from whole blood and subjected to RT-PCR using primers specific for EBOV GP. Samples were combined from 5 mice/ PI time point and run in triplicate for each experimental trial. For quantitative assessments, the average of the triplicate GE per reaction were determined and multiplied by 800 to obtain GE/mL plasma. Standard curves were generated using synthetic RNA. Error bars represent standard deviation. The limit of detection for this assay is 1 × 102 GE/mL of plasma (dotted line)

PBMC samples for this study were collected from control (Day 0) and five post-infection (PI) time-points (Days 1, 2, 3, 4 and 5) in two independent trials (Table 1). A serial sacrifice design was employed to ensure that adequate blood volume was collected at each PI time-point to provide sufficient numbers of PBMCs to support the proteomic endpoints. All animals in EBOV challenge groups (Groups 2–6) were infected with MaEBOV via the intraperitoneal (IP) route. MaEBOV infection recapitulates many facets of human EVD and has been used as a preclinical model to evaluate the efficacy of antiviral therapeutics, vaccines, and to investigate EBOV pathogenesis in the context of the mouse immune system [16, 17]. Blood samples pooled from animals at the PI time-points were used to determine the viral load by RT-PCR and purify PBMCs (see Fig. 1). After purification of PBMCs from each PI time-point, lysates were prepared in RIPA buffer (which inactivated the virus), samples were removed from bio-containment. Thirty micrograms of total protein from each PI time point was processed using filter-assisted sample prep (FASP), which included reduction/alkylation, Trypsin/Lys-C digestion and six-plex TMT labelling. The labelled peptides from each time-point were combined and analyzed in a single LC–MS/MS run. This work-flow provided semi-quantitative information on protein abundance in PBMCs during EBOV infection in mice.

### Protein quantification

Once combined and fragmented by MS/MS, each cumulative peptide spectrum contains a record of the abundance of that peptide from each time point. Proteins identified in each sample were analyzed for changes in abundance as compared to Day 1 (trial 1) or Day 0 (trial 2). Day 1 was used as a baseline for the first trial as the Day 0 sample did not produce adequate total protein levels. The ratios of the intensity of reporter ions associated with the PI time points versus the intensity of the same reporter ion in the pre-infection sample were reported as relative peptide abundances. Peptide abundances across an individual protein or protein group are averaged to produce protein abundances. Protein abundances were considered significant at any PI time point if ratios were twofold greater than (peptide ratio of 2.0) or less than (peptide ratio of 0.5) the baseline sample for that trial. EBOV peptide data accumulated in previous Ebola NHP studies conducted at U.S. Army Medical Research Institute of Infectious Diseases (USAMRIID) provided a standardized peptide inclusion list for major viral proteins. The inclusion list works by monitoring each full MS scan for the presence of any list masses that are at least 1% of the base peak intensity (see Table 2).

In the event none of the masses are observed, the instrument continues with the top-15 data-dependent scans. The inclusion list method becomes particularly important with complex biological matrices such as PBMC lysates. An additional increase in sensitivity can be accomplished by simultaneously excluding highly abundant peptide ions that are repeatedly sampled due to chromatographic persistence, in some cases accounting for 40% of the entire peptide complement (e.g. hemoglobin and albumin). In this study a combination of doubly- and triply-charged masses from 5 peptides of hemoglobin (Additional file 1: Table S1) were used to create an exclusion list which resulted in a 7% improvement in total quantified proteins (834 vs 906). The host proteins identified from the PBMC samples that change expression during EBOV infection are being evaluated and will be reported in a future publication.

### Viral RNA and viral protein detection

**Fig. 2:**
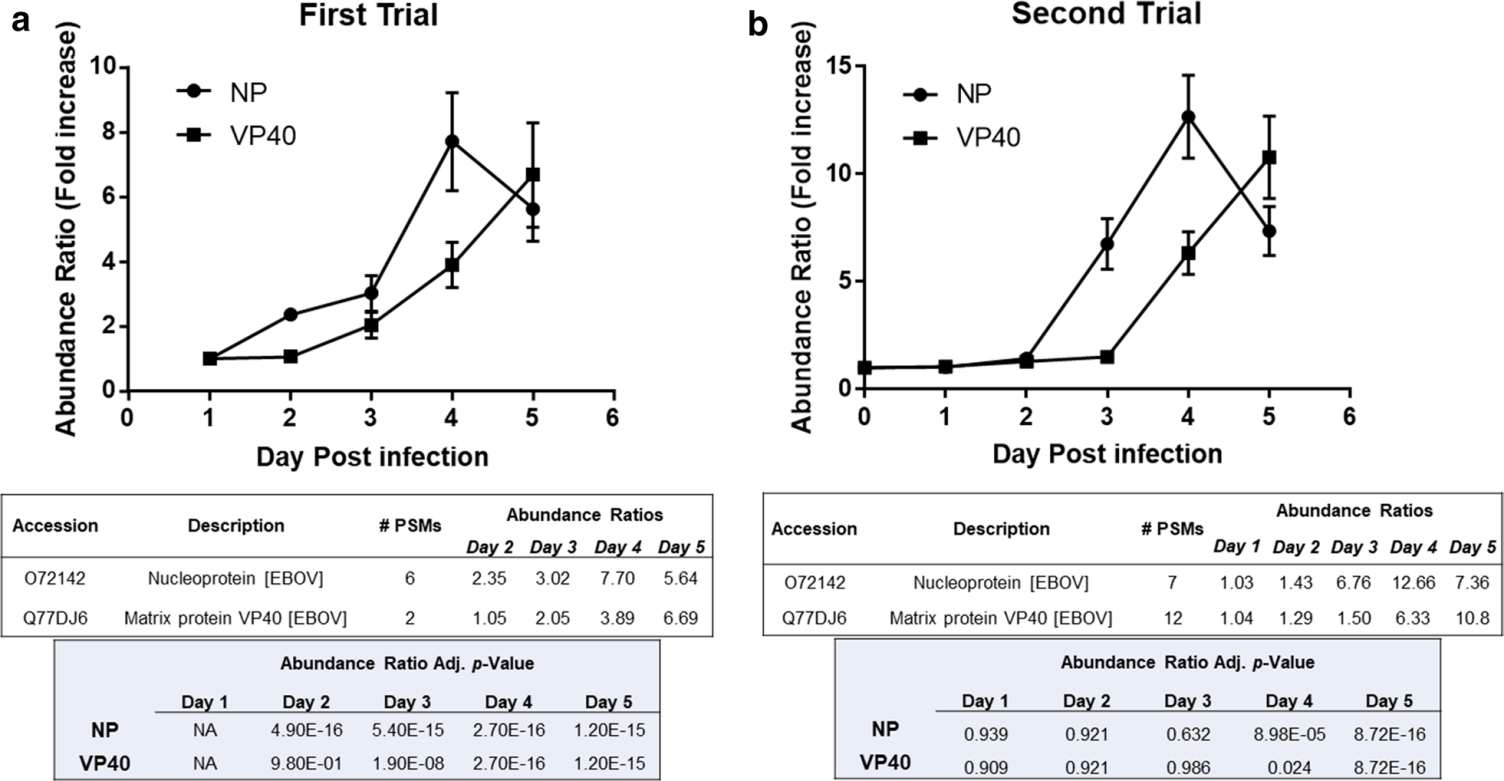
Detection and quantitation of EBOV NP and VP40 in PBMCs harvested from MaEBOV infected mice. Abundance changes of MaEBOV proteins NP and VP40 over 5 days of infection for Trial 1 (**a**) and Trial 2 (**b**). TMT 6-Plex quantitation data was merged from a total of five targets. Error bars represent the abundance ratio CV%. Tables below each graph display the abundance ratios (fold-change values from baseline) and statistics obtained for each protein quantitation on each day PI

**Fig. 3:**
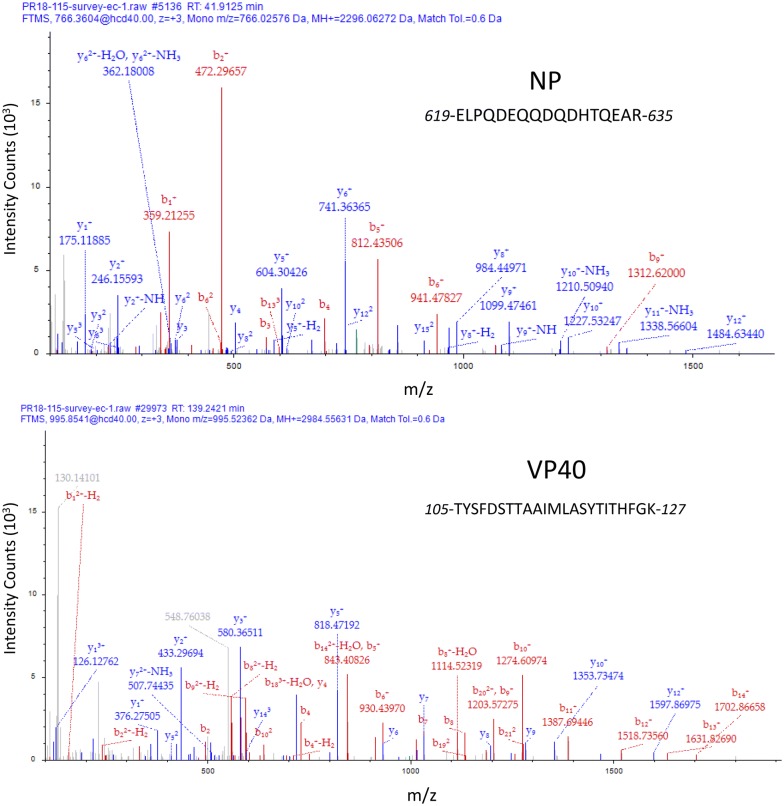
Representative MS/MS spectra and ion assignments for EBOV peptides attributed to NP (top) and VP40 (bottom) detected in PBMCs. Each series contains at least 10 contiguous y-ion fragments as well as Percolator PEP scores < 10^−5^ indicating a high confidence sequence match

As shown in Fig. 1, viral RNA was detected via RT-PCR in the EBOV infected mouse blood beginning on Day 2 PI and reached a maximum of 1.13E+07 genomic equivalents/mL (GE/mL) ± 10% on Day 5 PI in both independent studies. The TMT LC-MS/MS quantitation results from both experimental trials are shown in Fig. 2. Since our experimental design provides both detection and relative quantitation information, we were able to detect an increase in the abundance of the matrix protein VP40 and NP in PBMC samples extracted on Day 2 PI in both trials. Sequence confirmation was obtained and representative MS/MS spectra for VP40 and NP peptides is provided in Fig. 3. As seen in Fig. 2, in Trial 1 NP was increased 2.35-fold from the Day 1 sample which was used as a baseline value for this trial. In Trial 2, NP was increased 1.43-fold on Day 2 when Day 0 was used as the baseline sample. For Trial 2, the Day 1 values for NP and VP40 are only slightly above baseline (1.03 and 1.04 respectively) indicating that the protein was present and detected in the sample. In both trials a maximum fold change for NP was reached on Day 4, (7.7-fold for trial 1 and 12.66-fold for trial 2), and then dropped off slightly at Day 5 PI. The matrix protein VP40 was detected in PBMC samples from both trials. On Day 3, PI VP40 levels were 1.29-fold higher than the pre-infection level for Trial 2, but were only slightly higher than baseline in Trial 2 (1.05-fold). For both trials VP40 levels were increased appreciably by Day 3 PI (twofold for trial 1- and 1.5-fold for Trial 2). A maximum fold change for VP40 was reached on Day 5 for both trials. For Trial 2, VP40 was increased 10.8-fold on Day 5 PI, whereas a fold-change increase of 6.67 was observed on Day 5 PI for Trial 1. This continual increase is in contrast to what was observed for levels of NP, which reached a maximum on Day 4 PI.

Statistical data obtained from Proteome Discoverer is also provided for the viral proteins detected in each Trial (Fig. 2). The variability of the fold change values of the reporter ions was lower for Trial 1 than for Trial 2 (data not shown) and therefore lower p-values were obtained. For Trial 1, significant p-values were obtained for the fold change values observed for NP beginning on Day 2 PI (*p* = 4.90E−16) through Day 5 PI (p = 1.20E−15). Similarly *p*-values obtained for the fold changes observed for VP40 in Trial 1 were significant beginning on Day 3 (*p* = 1.90E−08) through Day 5 PI (*p* = p = 1.20E−15). Although the variability in the abundance ratios were higher for trial 2 the fold-change increases observed for NP and VP40 were significant on Days 4 and 5 PI.

### Western blot confirmation of EBOV VP40 and NP in mouse PBMC lysates

**Fig. 4:**
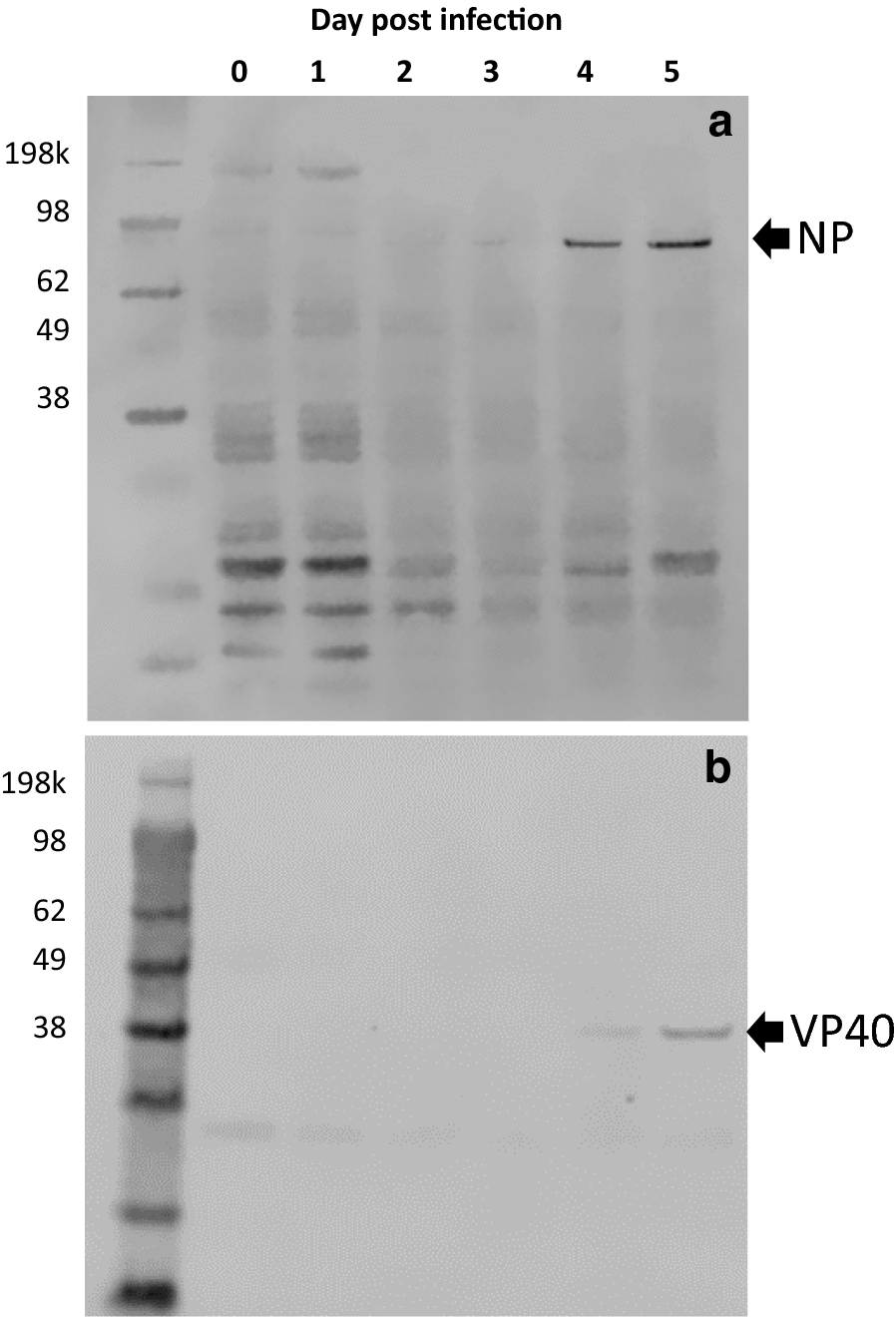
Western blot confirmation of the presence of EBOV NP (**a**) and VP40 (**b**) in PBMCs. Thirty micrograms of PBMC lysate from each pooled time point from Trial 2 and was run on a 4–12% gel under reducing conditions. The far-left lane contains the molecular weight marker. Arrows indicate immuno-specific bands for NP (**a**) and VP40 (**b**)

To provide orthogonal confirmation of the detection and quantitation of EBOV proteins NP and VP40, western blot was performed on PBMC lysates (30 µg) from each time point from Trial 2. As shown in Fig. 4a, NP was detected via western blot beginning on Day 3 PI and increased in abundance through Day 4 and Day 5 PI. The NP antibody used for this western blot was polyclonal and non-specific bands appear in all lanes. The non-specific band located around 200kDa may be CD45, a ubiquitous membrane protein of lymphocytes with a molecular mass of about 200 kDa [18]. This non-specific band is reduced in later post-infection days, indicating that the protein components of PBMC are altered during infection. The EBOV protein VP40 was detected by Day 4 PI and increased in abundance on Day 5 PI (see Fig. 4b). Detection of NP and VP40 by western blot occurred at least 1 day later than what was observed with mass spectrometry, but confirms the presence and increased expression of these two viral proteins in PBMC samples using an orthogonal detection method.

## Discussion

MaEBOV strains cause lethality in BALB/c, C57BL/6 and CD-1 mice, with death usually occurring between days 4–6 PI. Like the wild-type EBOV in the NHP model, MaEBOV infection produces a robust pro-inflammatory cytokine response, leading to extensive organ damage and wide-spread lymphocyte apoptosis [19–22]. Gibb et al reported that in BALB/c mice, symptoms did not appear until Day 3 PI (ruffled fur, weight loss), and both viral RNA (in-situ hybridization) and proteins (VP40, GP via immunohistochemistry) were detected in lymph nodes by Day 2 PI [21]. In both NHP EBOV infection and mouse MaEBOV infection, dendritic cells, monocytes and macrophages are the initial target cells for viral replication and dissemination throughout the body of the host [11, 12]. Therefore, the mouse model is particularly useful when studying the early stages of EBOV infection and the role of immune mediators produced by cells of the mononuclear phagocytic system to clearance and/or pathogenesis of the virus. Pre-symptomatic detection of EBOV proteins from blood or tissues has not been previously published, however, Sanchez et al detected EBOV soluble GP (sGP) and GP via western blot from glycoproteins purified from the serum of patients in the acute stages of infection [23]. Additionally, Rowe et al. detected EBOV antigens 3–6 days after symptom onset using ELISA [24]. The ReEBOV immunoassay platform detects VP40 in plasma and whole blood and can be useful to support a provisional diagnosis based on clinical exam and exposure history. However, ReEBOV testing failed to detect patients who were early in the disease course, and evaluation of ReEBOV in a field setting found that it only detected ~ 90% of cases that were positive by RT-PCR [25, 26]. In a laboratory setting, the lower limit of detection for ReEBOV in blood samples from infected macaques was 3 × 10^5^ GE/mL [27]. Detection of EBOV proteins by untargeted LC–MS/MS in plasma or tissues from infected humans or animals has not been reported, but we have detected sGP, GP and VP40 in EBOV infected NHP plasma (10 µl sample) by Day 4 PI in high titer samples (i.e. > 10^7^ GE/mL: data not reported). Clearly, alternative sample types, sample enrichment methods and more sensitive platforms are needed to improve protein antigen detection for diagnosis in a field setting. Commercial serological tests have been developed that target IgM and IgG elicited following exposure to EBOV [4, 28]. However, these tests are only reliably predictive of EVD long after the onset of clinical symptoms, and significantly after RT-PCR and viral antigen based tests are shown to provide positive diagnostic results. Therefore, the utility of tests that assess antibody responses to pinpoint EBOV as etiologic agent of an infection are limited especially at pre-symptomatic and/or acute stage of EVD. EBOV IgM has a relatively shorter half-life compared to other viral IgM classes based on analysis of samples obtained from EVD survivors further limiting its diagnostic window [2].

PBMC sample collection has been used historically for the detailed characterization of immune responses to infectious agents. These leukocytes are isolated from freshly drawn whole blood and comprise monocytes, lymphocytes (both B and T cells), and a small percentage of other immune cells, such as dendritic cells. PBMC function and characterization can be evaluated down-stream via re-stimulation of the cells in culture and flow-cytometry to determine cell type, viability and proliferation. The use of PBMC sample collection for diagnostic purposes for the detection of viral proteins or nucleic acids has also been performed using flow-cytometry techniques and RT-PCR [29–32]. However, no study to date has sampled EBOV infected PBMC at various times PI to determine temporal based analysis for diagnostic potential by targeting viral proteins.

In this study we collected PBMC samples from MaEBOV infected B57BL/6 mice. These mice had detectable viral RNA (for EBOV GP) by Day 2 PI in plasma, and a maximum titer (10^7^ GE/mL) was reached by Day 5 PI. Our quantitative mass spectrometry technique detected the viral proteins VP40 and NP in PI samples collected on Day 2 PI and significant increases in the abundance of both proteins was observed from Day 2-Day 5 PI. Several factors likely contribute to the early detection of VP40 and NP in PBMC lysates from mice infected with MaEBOV. VP40 is the most abundant viral protein located under the viral envelope, while GP is the most abundant protein on the viral surface. After viral fusion, transcription, and replication in a host cell, assembly of viral particles begins with the formation of nucleocapsids containing NP and viral RNA. Until an infected cell is lysed or a new virus buds, NP and VP40 remain intracellular. These nucleocapsids accumulate in the perinuclear region of the cell and are transported to budding sites at the plasma membrane. At the core of the nucleocapsid, NP helices are thought to interact physically with VP40 [33]. In the EBOV GP gene, there is an alternative transcription editing site which leads to the expression of sGP, and small sGP (ssGP), both of which are secreted [34]. These secreted forms of GP have been detected in the serum of infected animals and human patients [35]. GP proteins are synthesized in the endoplasmic reticulum (ER) and transported along the classical pathway of secretory proteins from the ER via the Golgi apparatus to the plasma membrane, most likely resulting in slower production of fully-processed GP when compared to NP or VP40. The extensive glycosylation of the GP can also hinder trypsin digestion, essentially providing fewer peptides for facile LC–MS/MS detection. Therefore, we posit that detection of VP40 and NP (but not GP) in the PBMC lysates at the early stages of infection is logical based on the protein properties (i.e. glycosylation, secretion of soluble forms) and the kinetics of the EBOV life-cycle.

Our study will facilitate the exploration of using PBMCs harvested from whole blood to develop a new fieldable immunoassay for the diagnosis of EVD at the earliest stages of infection. Genetic drift and the rapid accumulation of mutations in the EBOV genome can lead to major problems with the accuracy of some current RT-PCR based EBOV diagnostic assays. This was determined via whole genome sequence analysis of the EBOV viral samples from the 2014 epidemic [6]. Unlike nucleic acid targets, immunoassays that target viral protein antigens are less susceptible to sequence erosion. Furthermore, antibodies developed against highly conserved viral regions of NP and VP40 should allow detection of multiple EBOV species using a single immunoassay. Several hurdles will need to be overcome in the development of this diagnostic paradigm. Studies designed to determine the limit of detection and minimum cell number required for reliable viral protein detection are needed. Additionally, the development of PBMC enrichment devices that do not require centrifugation is crucial. The expected yield of PBMC from 1 mL of adult human blood is 0.8 to 3.2 × 10^6^ cells/mL and murine totals are similar, although the percentages of individual cell types varies slightly [36, 37]. Therefore, in this study, the PBMC yield from 5 mL of pooled mouse blood (approx 1 mL collected from 5 mice/time-point) would be 4–16 × 10^6^ cells/mL. In this study, we used 30 µg of total protein from each PBMC lysate as input material for the FASP digestion and TMT labeling. This was essentially enough material for approximately 40 LC-MS/MS runs. This bodes well for improving the sensitivity of detection using much lower whole blood volumes. Future studies will explore the use of less than 1 mL of blood for PBMC harvest/lysis and downstream LC–MS/MS.

## Conclusions

In conclusion, we interrogated PBMC samples obtained from MaEBOV exposed mice using a quantitative proteomic platform and demonstrated the presence of detectable levels of EBOV proteins before onset of clinical symptoms. The similarity between MaEBOV mice and NHP models in regard to EVD progression enabled us to perform cost-effective EBOV exposure study and obtain proteomic data. The proteomics result show that viral proteins are present at detectable levels in PBMCs at an earlier time-point after EBOV exposure compared to current detections capabilities provided by RT-PCR methods and immunoassays that target viral antigens in serum/plasma samples. RT-PCR is the most sensitive approach for EBOV detection but the method requires an RNA extraction step and the high rate of viral genomic mutations renders RT-PCR based assays less reliable during new viral strain outbreaks. Targeting viral proteins in PBMCs could provide invaluable diagnostic potential especially prior to clinical symptoms and before EVD is considered communicable. Viral protein enrichment in PBMC in tandem with highly sensitive immunoassays systems, such as electrochemiluminescent based detections, could lead to the development of a rapid, high-throughput automated diagnostic platform. In the future, investigations are required to: (i) develop rapid point-of-care PBMC enrichment techniques; (ii) assess compatibility of whole blood samples in immunoassay format to minimize sample processing steps and (iii) assess abundance of EBOV proteins in PBMC samples to estimate time of EBOV exposure. We envision that the interrogation of PBMC samples by proteomic analysis could potentially lead to the identification of detectable levels of bacterial and/or viral proteins indicative of infections by pathogens that are currently difficult to diagnose.

## Supplementary information

**Additional file 1: Table S1.** List of exclusion list masses commonly observed hemoglobin peptides.

## Data Availability

The datasets used and/or analyzed during the current study are available from TheProteomeXchange (PX) Consortium of proteomics resources (http://www.proteomexchange.org) and archived through the MassIVE data repository. (https://massive.ucsd.edu/)

## Abbreviations

EBOV: Ebola virus
EVD: Ebola virus disease
FASP: Filter assisted sample prep
GE: Genomic equivalents
HCD: Higher energy collisional dissociation
IP: Intraperitoneal
LC–MS/MS: Liquid chromatography tandem mass spectrometry
MaEBOV: Mouse-adapted Ebola virus
MARV: Marburg virus
NHP: Non-human primate
PBMC: Peripheral blood mononuclear cell
PFU: Plaque-forming units
PSM: Peptide spectrum match
RNA: Ribo-nucleic acid
RT-PCR: Real-time polymerase chain reaction
TMT: Tandem mass tags
