# Supplementary material for: Early detection of Ebola virus proteins in peripheral blood mononuclear cells from infected mice
Source: Clin Proteomics. 2020 Mar 17;17:11. doi: 10.1186/s12014-020-09273-y (PMC7077124; doi:10.1186/s12014-020-09273-y)
Supplement: Supplementary file 1 — Additional file 1: Table S1. List of exclusion list masses commonly observed hemoglobin peptides. [file 12014_2020_9273_MOESM1_ESM.pdf]

Peptide	m/z
DFTPAAQAAFQK	584.9929
DFTPAAQAAFQK	876.9871
GTFASLSELHCDK	481.507
GTFASLSELHCDK	641.6708
GTFASLSELHCDK	962.0054
lLVVYPWTQRYF	501.9672
LLVVYPWTQRYF	752.4458
VITAFNDGLNHLDSLK	554.5674
VITAFNDGLNHLDSLK	739.0875
VITAFNDGLNHLDSLK	1108.127
YFDSFGDLSSASAIMGNAkV	610.5621
YFDSFGDLSSASAIMGNAkV	813.7452
YFDSFGDLSSASAIMGNAkV	1220.114
